# Gamma delta T cells and their immunotherapeutic potential in cancer

**DOI:** 10.1186/s40364-025-00762-6

**Published:** 2025-03-28

**Authors:** Stephen G. Cieslak, Reza Shahbazi

**Affiliations:** 1 Division of Hematology/Oncology, Department of Medicine, Indiana University, Indianapolis, IN USA; 2 Department of Biochemistry and Molecular Biology, Indiana University, Indianapolis, IN USA; 3 Tumor Microenvironment & Metastasis, Indiana University Melvin and Bren Simon Comprehensive Cancer Center, Indianapolis, IN USA; 4 Brown Center for Immunotherapy, Indiana University, Indianapolis, IN USA

**Keywords:** γδ T cells, γδ T cell receptors, Immunotherapy, Cancer, Immunology, Oncology, CAR

## Abstract

Gamma–delta (γδ) T cells are a unique subset of T lymphocytes that play diverse roles in immune responses, bridging innate and adaptive immunity. With growing interest in their potential for cancer immunotherapy, a comprehensive and inclusive exploration of γδ T cell families, their development, activation mechanisms, functions, therapeutic implications, and current treatments is essential. This review aims to provide an inclusive and thorough discussion of these topics. Through our discussion, we seek to uncover insights that may harbinger innovative immunotherapeutic strategies. Beginning with an overview of γδ T cell families including Vδ1, Vδ2, and Vδ3, this review highlights their distinct functional properties and contributions to anti-tumor immunity. Despite γδ T cells exhibiting both anti-tumor and pro-tumor activities, our review elucidates strategies to harness the anti-tumor potential of γδ T cells for therapeutic benefit. Moreover, our paper discusses the structural intricacies of the γδ T cell receptor and its significance in tumor recognition. Additionally, this review examines conventional and emerging γδ T cell therapies, encompassing both non-engineered and engineered approaches, with a focus on their efficacy and safety profiles in clinical trials. From multifunctional capabilities to diverse tissue distribution, γδ T cells play a pivotal role in immune regulation and surveillance. By analyzing current research findings, this paper offers insights into the dynamic landscape of γδ T cell–based immunotherapies, underscoring their promise as a potent armamentarium against cancer. Furthermore, by dissecting the complex biology of γδ T cells, we learn valuable information about the anti-cancer contributions of γδ T cells, as well as potential targets for immunotherapeutic interventions.

**Graphical abstract:**

Gamma–delta T cell in action.

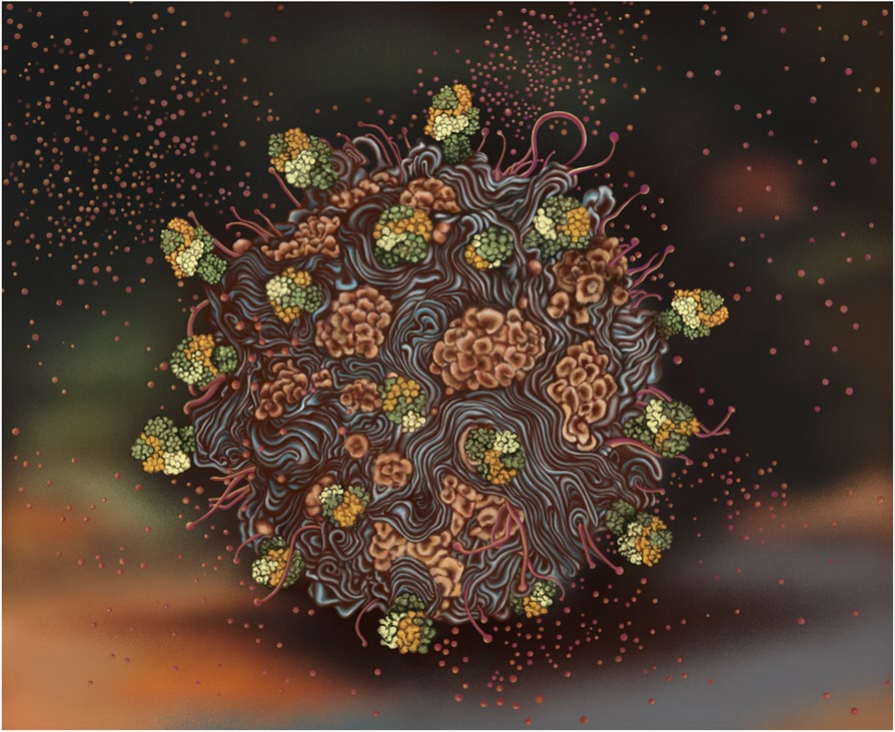

## Introduction

Gamma–delta (γδ) T cells, a unique subset of T lymphocytes, have emerged as pivotal players in the orchestration of immune responses, wielding a diverse array of functions that extend beyond conventional alpha–beta (αβ) T cells. With an unconventional T cell receptor (TCR) repertoire and distinct tissue distribution, γδ T cells serve as versatile guardians of immunity, bridging innate and adaptive immune responses. While other reviews discuss various aspects of γδ T cells and their roles in cancer immunotherapy, our review article provides a comprehensive, inclusive overview of γδ T cell families and subtypes, development, activation, functions, therapeutic implications in cancer immunology, and structure, as well as current therapies developed in academic, clinical, and corporate settings.

For instance, Vδ1 T cells are important for anti-tumor immunity and play a role in responding to solid tumors through cytotoxic activity and recognition of both peptide and non-peptide antigens. Vδ2 T cells, mainly located in the blood, activate anti-tumor immunity in cancers typically by responding to phosphoantigens, while Vδ3 T cells respond to oxidative stress markers in cancer, as well as both traditional and non-traditional antigens. Through elucidating the mechanisms governing γδ T cell biology and the intricacies of each γδ T cell family, we seek to unveil novel insights that may pave the way for innovative immunotherapeutic strategies and interventions.

While γδ T cells have shown great promise in preclinical and clinical studies, their role in cancer remains complex due to their ability to exert both beneficial and detrimental effects on tumor progression. This dual functionality has led to challenges in optimizing γδ T cell–based therapies. The ability of γδ T cells to recognize and respond to tumor-associated antigens opens new avenues for cancer treatment, particularly when engineered or activated to enhance their anti-tumor potential. Overall, this review delves into conventional and novel approaches to γδ T cell therapy, including engineered therapies, focusing on their therapeutic efficacy and safety in clinical trials. By synthesizing current research, this review highlights the growing potential of γδ T cells as a powerful tool in the fight against cancer.

## γδ T cell biology

Γδ T cells and αβ T cells develop from common thymic progenitors but differ in various functional aspects, such as faster activation and reduced propensity of γδ T cells to induce graft-versus-host disease (GvHD). Unlike αβ T cells, γδ T cells do not rely on traditional major histocompatibility complex (MHC)-mediated antigen presentation, and some subsets show adaptive responses similar to αβ T cells. For example, Vδ1 T cells, largely found in peripheral tissues, play critical roles in tissue balance and immunity, exhibiting cytotoxic activities through natural killer receptors (NKRs) , particularly in cancer contexts like multiple myeloma and colorectal cancer. Vδ2 T cells, primarily present in the blood, are involved in anti-tumor immunity and can be activated by phosphoantigens or aminobisphosphonates, promoting cytotoxicity in cancers such as multiple myeloma and renal cell carcinoma. Vδ3 T cells are functionally and locationally similar to Vδ1 T cells; however, Vδ3 T cells can secrete helper T cell cytokines and play a role in oxidative stress recognition in cancer settings. Moreover, γδ T cells demonstrate both anti-tumor and pro-tumor properties, with roles in immune surveillance and cancer progression, potentially offering novel therapeutic avenues through their innate receptor activation, bypassing traditional antigen-specific mechanisms.

**Fig. 1:**
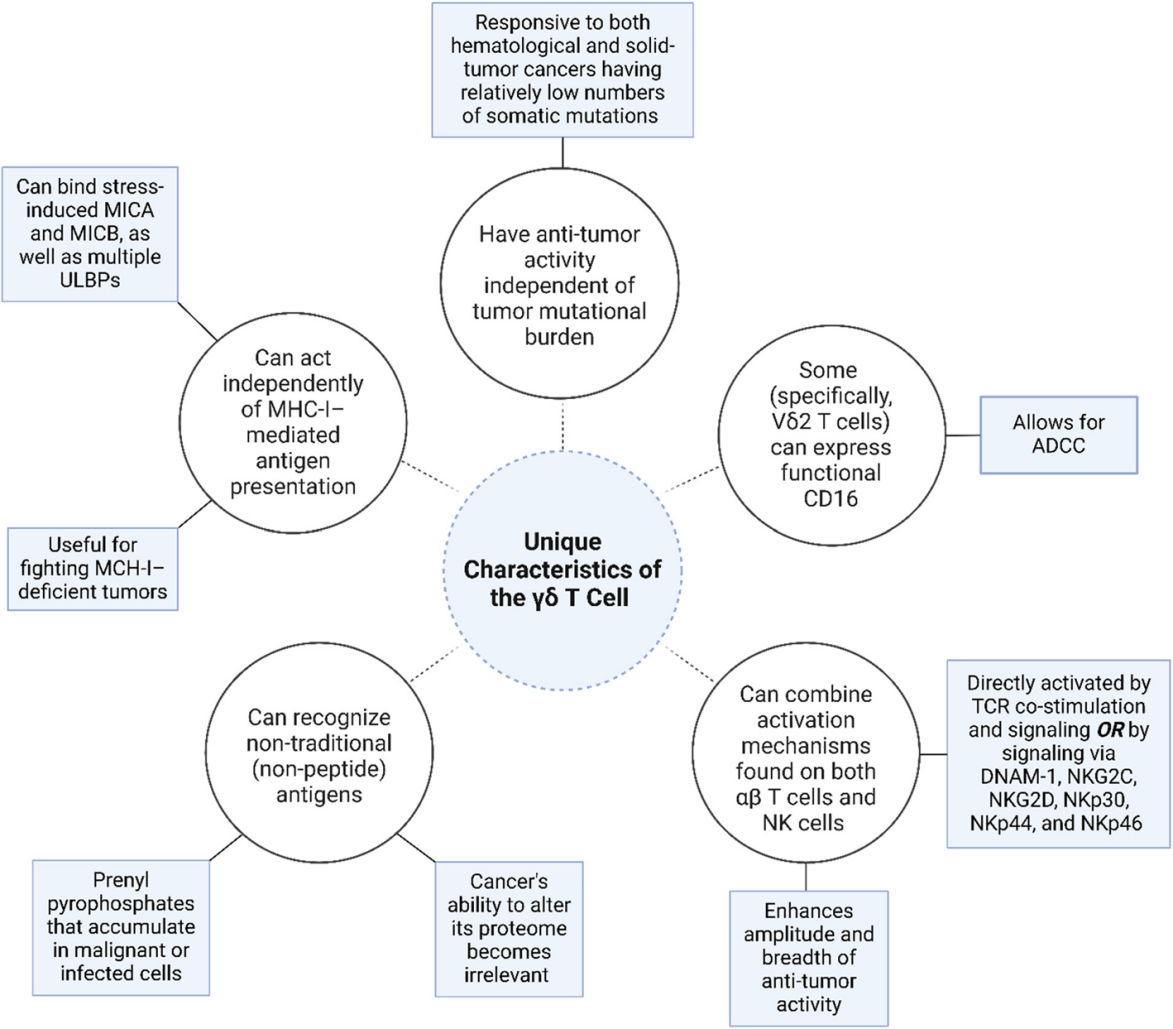
The unique characteristics of γδ T cells. γδ T cells are unique in that γδ T cells can act independently of MHC-I–mediated antigen presentation, have anti-tumor activity independent of tumor mutational burden, combine activation mechanisms found on conventional T cells and NK cells, recognize non-traditional antigens, and express functional CD16, allowing for ADCC. ADCC, antibody-dependent cellular cytotoxicity; CD, cluster of differentiation; DNAM-1, DNAX accessory molecule 1; NKG2C, natural killer group 2C; NKG2D, natural killer group 2D; MHC-I, major histocompatibility complex 1; MICA, MHC class I chain–related protein A; MICB, MHC class I chain–related protein B; ULBP, UL-16 binding protein; NK, natural killer; TCR, T cell receptor; NKp, natural killer protein. Created with BioRender.com

Although γδ T cells and αβ T cells develop from shared thymic progenitors, γδ T cells exhibit fundamental differences from αβ T cells (Fig. 1), such as faster functional activation at peripheral sites and a markedly reduced tendency to induce GvHD [1]. Moreover, γδ T cells do not rely on classic MHC-mediated antigen presentation and can even normally develop without MHC-II (Fig. 1) [1–3]. Additionally, γδ T cells are fundamentally similar to natural killer (NK) cells in that γδ T cells recognize neoplastic or infected cells through multiple receptor–ligand interactions, quickly responding in an innate-like manner without requiring prior exposure [1, 4, 5]. However, a subset of γδ T cells have been observed to clonally expand during primary infections and effectuate adaptive immune responses upon subsequential challenge, resembling the behavior of αβ T cells [1, 6]. Furthermore, in the intestines, γδ T cells exhibit protective and multifunctional memory [6]. Consequently, these characteristics position γδ T cells at the interface between αβ T lymphocytes and NK cells (Fig. 1) [1].

### Vδ1 T cell biology

**Fig. 2:**
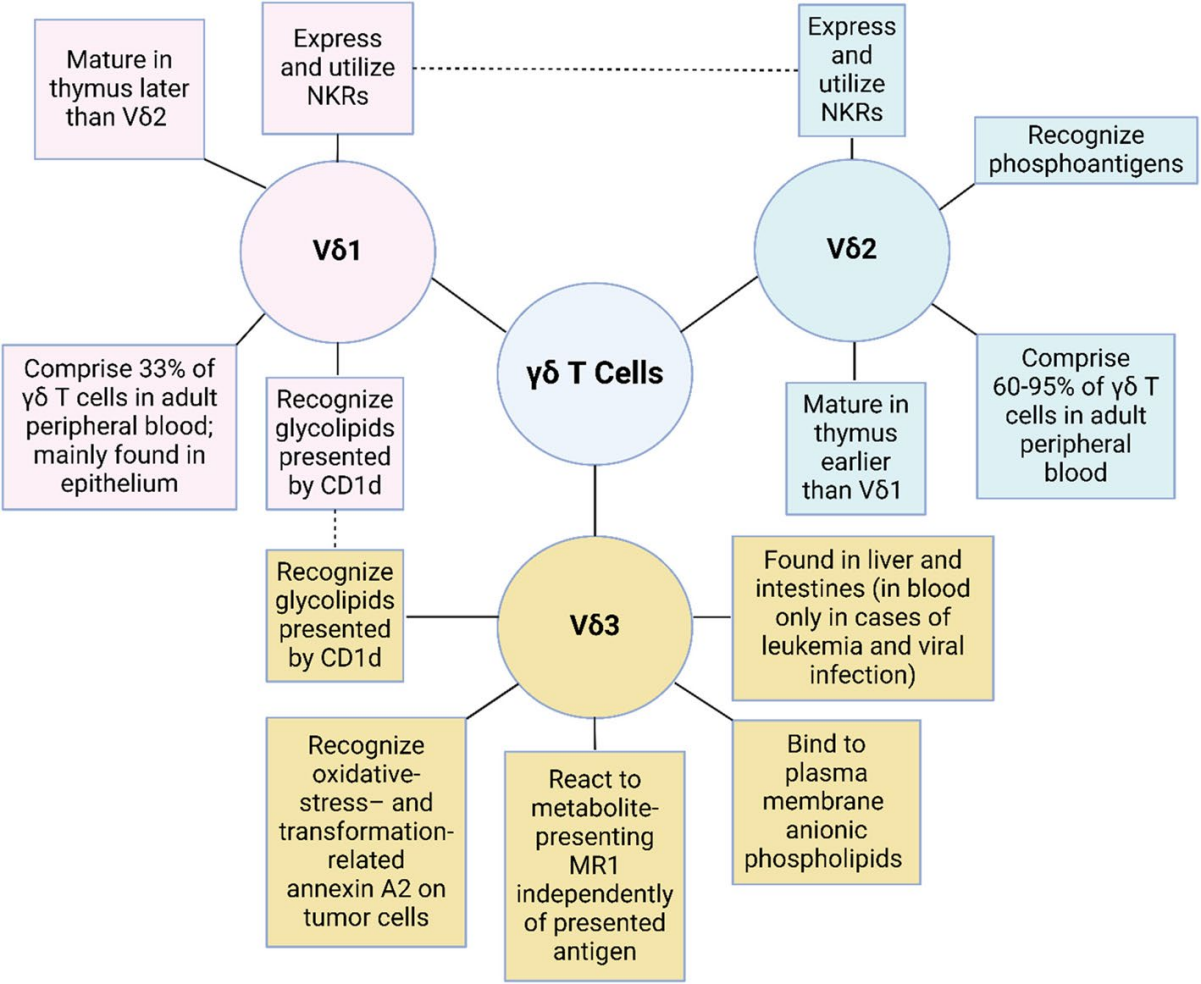
The differences between γδ T cell families. γδ T cells have three functional families: Vδ1, Vδ2, and Vδ3. Vδ1 T cells are mostly tissue-resident, making up only 33% of γδ T cells in the peripheral blood. Moreover, Vδ1 T cells mature in the thymus later than Vδ2 T cells. Vδ2 T cells are mostly circulatory, comprising 65–90% of γδ T cells in the peripheral blood. These γδ T cells are known for their ability to recognize phosphoantigens. Both Vδ2 and Vδ1 T cells can express and utilize NKRs. Vδ3 T cells are not well known but are mainly found in the liver and intestines responding to cellular metabolites and oxidative stress markers. However, in cases of leukemia and viral infection, Vδ3 T cells can be found in the peripheral blood. Notably, both Vδ3 and Vδ1 T cells can recognize glycolipids presented by CD1d. Dotted lines connect common characteristics between two γδ T cell families. CD, cluster of differentiation; NKR, natural killer receptor; MHC-I, major histocompatibility complex 1; MR1, MHC class I–related protein 1. Created with BioRender.com

**Fig. 3:**
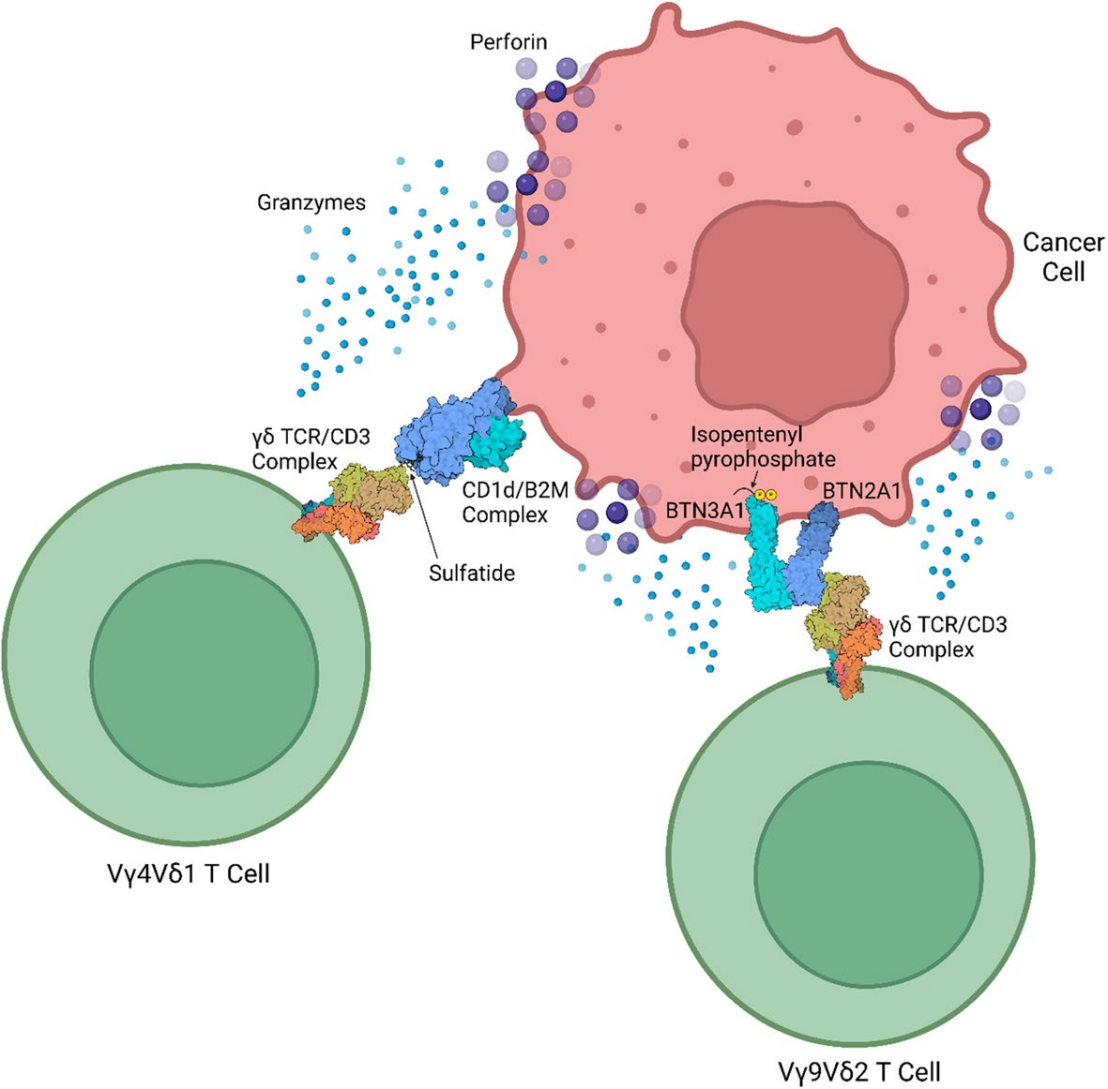
Common γδ T cells interacting with cancer cell. Phosphoantigens, such as isopentenyl pyrophosphate, do not directly interact with Vγ9Vδ2 TCRs but instead bind to the BTN3A1 intracellular domain, thus allowing BTN3A1 to interact with BTN2A1, which can then bind to the Vγ9 chain. On the other hand, Vγ4Vδ1 TCRs can bind to sulfatide – a glycolipid presented by CD1d. BTN3A1, butyrophilin subfamily 3 member A1; BTN2A1, butyrophilin subfamily 2 member A1; CD, cluster of differentiation; TCR, T cell receptor; B2M, β_2_ microglobulin. Created with BioRender.com

Vδ1 T cells are produced in the human thymus after Vδ2 T cells (Fig. 2), typically appearing around 4–6 months after birth [1, 7]. While Vδ1 T cells make up only about one-third of circulating γδ T cells in healthy adults (Fig. 2), Vδ1 T cells are predominantly found in various peripheral tissues such as the liver, gut epithelium, spleen, and dermis, playing crucial roles in maintaining tissue balance [1, 8]. In intraepithelial gut tissue, Vγ4Vδ1 cells bind BTNL3 and endothelial protein C receptor (EPCR), a stress-induced MHC-I–like molecule, via complementary-determining region 3 of the Vγ4 chain, where Vγ4 binds to BTNL3 with 3.6–sixfold higher affinity than to EPCR [9–12]. While Vγ4–EPCR binding can be inhibited by the markedly higher binding affinity of Vγ4 to BTNL3, BTNL3 is significantly downregulated in colorectal cancer (CRC) whereas EPCR is commonly overexpressed in CRC and multiple other cancers [10–17]. On the other hand, some Vδ1 T cell subtypes, such as Vγ2Vδ1 and Vγ3Vδ1, do not bind to BTNL3 [11]. Other Vδ1 T cell subtypes, such as Vγ5Vδ1 and Vγ4Vδ1, can bind to sulfatide, a glycolipid presented by CD1d (Fig. 3) [18]. Moreover, a non-prevalent and lesser-known Vδ1 T cell subtype, Vγ9Vδ1, can bind EPHA2 once EPHA2 is coordinately recognized by ephrin-A [19]. Interestingly, EPHA2 expression is upregulated during AMP-activated protein kinase (AMPK)-dependent metabolic cancer cell reprogramming and co-expression of AMPK and EPHA2 in CRC tumors is correlated with higher CD3^+^ T cell infiltration [19]. Vγ9Vδ1 TCRs can also bind distal to the metabolite binding cleft of the metabolite-presenting MHC class I–related protein 1 (MR1) [20].

Akin to Vδ2 T cells, Vδ1 T cells express innate NKRs (Fig. 2) [1]. For instance, when natural killer group 2D (NKG2D) is engaged by ligands like MHC class I chain–related protein A (MICA) and UL-16 binding proteins (ULBPs), Vδ1 T cells release perforin and granzyme B, with DNAX accessory molecule 1 (DNAM-1/CD225) also contributing to Vδ1 cytotoxic function [1, 21–24]. This phenomenon is especially observed in the strong response of Vδ1 T cells against primary multiple myeloma cells [23]. Another example is when Vδ1 T cells from B-cell chronic lymphocytic leukemia patients bind ULBP3 expressed on leukemia cells and are subsequently upregulated by trans-retinoic acid [24]. In specific contexts, Vδ1 T cells express NKRs from the natural cytotoxicity receptor family, such as NKp46 in the intestinal epithelium, enhancing Vδ1 cytotoxicity against CRC cells [1, 25]. Moreover, stimulation of Vδ1 T cells with IL-2 or IL-15 in vitro induces NKp30 and NKp44, augmenting Vδ1 anti-tumor activity against various leukemias [1, 21, 26]. Furthermore, in the clinical context of CRC samples lacking MHC-I and mismatch repair mechanisms, abundant Vδ1 and Vδ3 T cells expressing PD-1 and NKRs (particularly NKG2D), which play crucial roles in the efficacy of immune-checkpoint inhibitors, have been observed [1, 27].

### Vδ2 T cell biology

In humans and in certain non-rodent species, the first γδ T cells formed are Vδ2 T cells (Fig. 2) [1, 7, 28]. These cells originate from the early rearrangement of the Vδ2 gene during fetal development, typically between weeks 5–6 in the fetal liver and weeks 8–15 in the fetal thymus [1, 7, 28]. However, over time and due to sequential infections during childhood, Vδ2 T cells expand and eventually constitute a significant portion (60–95%) of γδ T cells in adult peripheral blood (Fig. 2) [1]. Interestingly, in the tumor microenvironment (TME), mevalonate pathway dysregulation can result in the accumulation of the lower-affinity phosphoantigen isopentenyl pyrophosphate (IPP), thus facilitating activation and chemotaxis of Vδ2 T cells [1, 29–32].

When IL-2 is present, stimulation by phosphoantigens results in the selective and swift expansion of Vδ2 T cells ex vivo, which exhibit anti-tumor activity in vitro, as is observed in cases of multiple myeloma and renal cell carcinoma [1, 33–36]. Moreover, IPP-activated CD56^+^ γδ T cells exhibit strong anti-tumor activity against squamous cell carcinoma (SCC) [37]. Furthermore, aminobisphosphonates like pamidronate and zoledronate have been clinically shown to promote Vδ2 activation and expansion in vivo, as well as Vδ2-mediated killing of multiple myeloma and chronic myelogenous leukemia cells [1, 38–41]. These compounds inhibit farnesyl pyrophosphatase synthase, interfering with the mevalonate pathway and leading to IPP accumulation, which facilitates Vδ2 T cell cytotoxicity and chemotaxis [1, 30, 31]. Recent studies propose using agonistic monoclonal antibodies targeting butyrophilin subfamily 3 member A1 (BTN3A1) to enhance Vδ2 T cell activation and cytotoxicity against tumor cells [1, 42–45]. However, whether diverse antigens can bind to the Vδ2 chain of the Vγ9Vδ2 TCR remains elusive [1]. Nevertheless, it has been suggested that various ligands binding to different regions of the same γδ TCR could enable γδ T cells to mediate both adaptive and innate responses [1, 9].

Apart from TCR-mediated recognition, Vδ2 T cells express surface NKRs that facilitate cytotoxic function upon recognizing ligands on target cells (Fig. 2) [1]. For example, the C-type lectin-like receptor NKG2D interacts with stress-induced MICA and MICB, in addition to multiple ULBPs, activating Vδ2 T cells [1, 46, 47]. Moreover, DNAM-1/CD225 interacts with nectin-2 and nectin-like 5/CD155, promoting hepatocellular carcinoma cell lysis [1, 48]. Additionally, these cells can express functional CD16, enabling antibody-dependent cellular cytotoxicity (ADCC) against tumor cells targeted with antibody-based therapies (Fig. 1), such as trastuzumab, which binds HER2 on breast cancers, and rituximab, which binds CD20 on B-cell lymphomas [1, 49–52]. Lastly, and contrary to other γδ T cells, Vδ2 T cells possess the ability to cross-present antigens similarly to dendritic cells, thereby inducing CD8^+^ αβ T cell responses [1, 53].

### Vδ3 T cell biology

Vδ3 T cells are rarely found in the peripheral blood of healthy individuals but are notable in the liver and intestines; nevertheless, Vδ3 T cells can be found in circulation during leukemia and viral infections (Fig. 2) [1, 54–57]. These γδ T cells share functional characteristics with Vδ1 T cells, such as the ability to bind glycolipids presented by CD1d (Fig. 2) [1, 58]. Notably, Vδ3 T cells can secrete T_H_1, T_H_2, and T_H_17 cytokines [58]. However, one Vδ3 TCR clone has exhibited reactivity to annexin A2 on tumor cells (Fig. 2), an intracellular protein that becomes upregulated during cellular transformation or oxidative stress, binds to anionic phospholipids in the cell membrane, and can be brought to the cell surface [1, 59]. Furthermore, certain Vδ3 TCR clones independently react to MR1 by binding atypical positions on or beneath the antigen-binding cleft of MR1 (Fig. 2) [1, 60, 61]. Some researchers have suggested an inherent MR1 autoreactivity of certain Vδ3 TCRs based on this unique mode of ligand recognition [1, 61]. Additionally, Vδ1^−^/Vδ2^−^ T cells have been identified in duodenal biopsy specimens and human blood that react with MR1 tetramers in a metabolite-independent manner (Fig. 2), indicating potential therapeutic implications [1, 61].

### Anti-tumor properties

Research on the roles of pleiotropic γδ T cells in human and mouse tumor immunology is extensive [1, 62, 63]. Similar to other T cell subsets, studies have emphasized the diverse functions of γδ T cells in the intestinal TME, showcasing a dynamic interplay between pro-tumor and anti-tumor γδ T cells [1, 63–67]. However, this functional duality has primarily been elucidated through mouse studies, where γδ T cells have a higher propensity than human γδ T cells to generate IL-17A, a cytokine associated with breast tumor metastasis and growth [1, 63, 68–70]. Contrarily, human γδ thymocytes inherently lean toward differentiating into γδ T cells upon stimulation with IL-2 or IL-15, and are consequently more inclined to exhibit anti-tumor effects within the TME [1, 71]. This process occurs through IL-2–mediated hyperphosphorylation of ERK1/2, AKT, and STAT5, where ERK1/2 is responsible for the differentiation of γδ thymocytes into IFN-γ^+^ or TNF-α^+^ γδ T cells [71]. It is also important to note that αβ T cells can facilitate γδ T cell function through the release of IL-2 and activation of the transcription factor T-bet [72].

Studies using syngeneic mouse models have clearly demonstrated the non-redundant role of γδ T cells in controlling prostate and spontaneous B-cell lymphoma tumor growth [1, 73–76]. Notably, the anti-tumor effector functions detected in mice are also manifest in human γδ T cells upon in vitro activation [1, 36, 37, 39–41]. These functions include the secretion of T_H_1 cytokines such as TNF-α and IFN-γ, the release of cytolytic granules containing granzymes A and B and perforin, and the expression of death receptor ligands like TRAIL and FASL, which can induce tumor cell apoptosis [1, 36, 37, 39–41]. Importantly, research has shown that IFN-γ secretion is inhibited by LAG-3, TIM-3, and PD-1, while TNF-α secretion is inhibited by TIM-3 [72, 77–81]. Moreover, B7-H3 on cancer cells can suppress IFN-γ expression in Vδ2 T cells by blocking T-bet [72, 82]. It is therefore plausible that at least some anti-tumor signaling pathways of γδ T cells work by inhibiting LAG-3, PD-1, and/or TIM-3; however, as discussed previously, it is known that αβ T cells can facilitate γδ T cell activity through the activation of T-bet [72]. Nevertheless, the TCR primarily determines γδ T cell activation and subsequent T_H_1 cytokine secretion, although NKRs are critical for the powerful cytotoxicity of γδ T cells (Fig. 1) [1, 83]. Akin to NK cells, NKG2D activation prompts γδ T cells to release cytolytic granules, leading to in vitro cytotoxic activity against various tumor cell types, including those derived from CRC, SCC, and human renal cell carcinoma [1, 22, 36, 37, 84]. NKG2D also regulates soluble TRAIL production in IL-2–activated human γδ T cells, which triggers apoptosis in lung cancer cell lines [1, 85]. Whether this mechanism extends to γδ T cells stimulated through other methods is uncertain [1]. Interestingly, whereas Vδ2 T cells can execute ADCC via CD16 expression (Fig. 1), Vδ1 T cells do not rely on CD16-dependent ADCC; instead, these cells mainly utilize granzyme B and perforin secretion to induce cytotoxicity, as has been demonstrated against neuroblastomas [1, 50, 51, 86].

In addition to directly targeting cancer cells, γδ T cells play a role in coordinating anti-tumor responses at various levels [1]. IFN-γ secretion by γδ T cells augments overall cytotoxicity by amplifying IFN-γ release by αβ T cells and increasing expression of MHC-I on cancer cells, which facilitate recognition of cancer cells by CD8^+^ T cells [1, 73, 87]. Furthermore, γδ T cells expressing CD137L/4-1BBL can co-stimulate NK cells, increasing ADCC and direct cytotoxicity against tumor cells [1, 88]. Additionally, Vδ2 T cells have the ability to cross-present MHC-I– and MHC-II–restricted peptides, functioning as antigen-presenting cells that efficiently trigger αβ T cell proliferation and activation, as well as induce peptide-specific CD8^+^ T cell responses [1, 89–91]. Previous opsonization of cancer cells with tumor-specific antibodies (also known as “licensing”) is necessary for this mechanism to occur [1, 92]. Notably, γδ T cells can also enhance antibody production by B cells in both humans and mice by inducing differentiation of T_FH_ cells [1, 93–97]. In humans, a subset of CXCR5-expressing Vδ2 T cells promotes antibody class switching and production through IL-4 and IL-10 secretion, as well as through (E)−4-hydroxy-3-methyl-but-2-enyl pyrophosphate (HMB-PP)-mediated upregulation of IL-21R [1, 93, 94]. Taken together, the diverse functions of γδ T cells highlight the significance of γδ T cells as anti-tumor effectors [1].

### Pro-tumor properties

In mouse models of cancer, studies have consistently elucidated the pro-tumor potential of IL-17–producing γδ T cells [1, 63, 68, 70, 98]. For example, mouse CD27^−^ γδ T cells that secrete IL-17A facilitate ovarian cancer growth by mobilizing pro-tumor small peritoneal macrophages, whereas IL-17–devoid γδ T cells are beneficial in ovarian cancer [98, 99]. However, in human peripheral blood, IL-17^+^ γδ T cells are nearly absent [1, 71]. Furthermore, a pan-cancer transcriptomic analysis that identified γδ T cells as strongly associated with a favorable prognosis found no correlation between γδ T cells and IL-17 expression in the TME [1, 100]. Nonetheless, other studies have reported observing tumor-infiltrating IL-17^+^ γδ T cells in CRC, SCC, HPV-associated uterine cervical SCC, and gallbladder cancer patients, particularly in advanced stages of the disease [1, 101–106]. This observation aligns with research describing temporal segregation and dynamic interplay between pro-tumor and anti-tumor intestinal γδ T cell subsets in humans and mice [1, 67]. These subsets, defined by enrichment for IL-17 expression and cytolytic markers, respectively, show that the pro-tumor subset amasses during CRC progression [1, 37, 66, 67].

IL-17–producing γδ T cells are responsible for several pro-tumor actions (especially in hepatocellular carcinomas and mammary cancers) including directly triggering malignant cell migration by upregulating MTA1 and proliferation by activating the IL-6/STAT3 signaling pathway, recruiting tumorigenic neutrophils, mobilizing polymorphonuclear myeloid-derived suppressor cells (PMN-MDSCs) to suppress anti-tumor cytotoxic activity of and IFN-γ production by Vδ2 T cells, increasing endothelial permeability, and facilitating vascular endothelial growth factor (VEGF)-dependent angiogenesis – all of which sustain an immunosuppressive TME [1, 104–113]. Interestingly, the expansion and activation of IL-17^+^ γδ T cells may be facilitated by commensal microbiota, as demonstrated in a lung adenocarcinoma mouse model [1, 114, 115]. Furthermore, disruption of the epithelial barrier in human CRC due to tumor growth allows bacterial invasion [1, 106]. Subsequent phagocytosis and antigen presentation by dendritic cells promote IL-17^+^ γδ T cell polarization, as well as production of IL-8 and granulocyte–macrophage colony-stimulating factor (GM-CSF), correlating with the TME-based accumulation of immunosuppressive PMN-MDSCs [1, 106]. Additionally, IL-17^+^ γδ T cells in mice facilitate neutrophil recruitment, in which neutrophils suppress pro-tumoral IL-17^+^ γδ T cells by inducing oxidative stress and establishing a multifaceted regulatory crosstalk between these immune populations [1, 68, 116–118]. This regulatory crosstalk, known as the “γδ T cell–IL17A–neutrophil axis” drives immunosuppression even to the point of conferring resistance to high-dose anti-VEGFR2 (vascular endothelial growth factor receptor 2) breast cancer therapy [118].

The possible pro-tumor functions of γδ T cells may not solely rely on IL-17 production [1, 119, 120]. Additional potential mediators include PD-L1, galectin 1, and galectin 9 which suppress effector T cells when interacting with PD-1 and glycosylated receptors, respectively [1, 119, 120]. Specifically, PD-L1 and galectin 9 expression by tumor-infiltrating γδ T cells have been shown to functionally exhaust and inhibit αβ T cells in pancreatic cancer mouse models [1, 119]. Moreover, pancreatic cancer patients were observed to have increased levels of PD-L1 and galectin 9 in circulating γδ T cells, with even higher levels in tumor-infiltrating γδ T cells, compared to healthy individuals [1, 119]. Another possible pro-tumor pathway involves IL-4 and IL-10 secretion, which is connected with Vδ1 T cells exhibiting a regulatory phenotype and inhibiting Vδ2 T cell function [1, 121, 122]. Regulatory γδ T cells can infiltrate human breast tumors and inhibit dendritic cell and αβ T cell activity, as well as suppress adaptive and innate immunity, potentially by inducing senescence [1, 123, 124]. However, interaction with Toll-like receptor 8 ligand in mice and in vitro reversed this effect [1, 123, 124]. Furthermore, in the breast cancer TME, a CD73-expressing Vδ1 T cell subset was detected, in which cells produce adenosine, IL-8, and IL-10, implying an immunosuppressive phenotype [1, 125]. Nevertheless, a recent study showed that γδ T cells, primarily Vδ1 T cells, that infiltrate triple-negative breast cancers produce an abundance of IFN-γ and are positively associated with overall survival and progression-free survival [1, 126]. These discoveries suggest that despite the existence of minor immunosuppressive γδ T cell subsets, these subsets are typically surpassed by anti-tumor γδ T cells [1]. Additionally, γδ T cells usually produce higher amounts of anti-tumor cytokines (such as TNF and IFN-γ) than pro-tumor cytokines (for instance: IL-4, IL-10, IL-17, IL-22, and IL-35) within the TME, reflecting their prognostic value in cancer patients [1, 102, 104].

### Therapeutic implications

Existing cancer immunotherapies heavily rely on the presence of tumor-associated antigens and/or neoantigens, which aligns with the functioning of B cells and αβ T cells [12, 127, 128]. Unfortunately, cancers inherently have significant epigenetic variability and genomic instability, leading to flaws in antigen presentation and/or the suppression of neoantigens [12, 129–135]. Consequently, immune evasion and resistance to current immunotherapies occur; however, neoantigens are not the sole pathway to immunological recognition in cancer [12, 129, 131–135]. While genomic instability may impede antigen-specific αβ T cell immunosurveillance, this instability also prompts immunological stress ligand expression on cancer cells like the MULT1/H60/RAE-1 families in mice and ULBP/MIC families in humans [12, 136, 137]. These ligands, which are regulated by the DNA damage response pathway, interact with the innate activating receptor NKG2D, which is expressed by γδ T cells, CD8^+^ αβ T cells, and NK cells [12, 136]. Additionally, γδ T cells express various other innate activating receptors like NKG2A, NKG2C, NKp30, NKp46, and DNAM-1 whose ligands are often present on stressed neoplastic cells [21, 25, 26, 48, 99, 138–141]. Interestingly, NKG2A inhibits NKG2C effector functions of Vδ2 T cells while conferring on Vδ2 T cells the highest anti-tumor effector functions [138, 140]. Moreover, recent research highlighted a unique subset of Vδ1 T cells in human intestinal epithelium expressing NKp30, NKp46, NKG2C, and NKG2D, with powerful NKp46-dependent cytolytic responses against CRC [12, 25]. Notably, the presence of Vδ1 T cells in CRC correlated significantly with lower-stage disease [12]. Furthermore, γδ T cells can be directly activated by innate receptors without requiring simultaneous antigen-specific TCR signaling, unlike most αβ T cells (Fig. 1) [12, 26, 126]. Nevertheless, γδ T cells can still be activated through the γδ TCR, although not via conventional MHC engagement but rather by detecting self-encoded molecules associated with cellular distress and tissue health [12]. Upon activation, these cells primarily generate anti-tumor cytokines like IFN-γ, release cytotoxic granules, and eliminate cancer cells [1, 12, 37]. Consequently, γδ T cells may offer cancer immunosurveillance without the use of antigen-specific adaptive αβ T cells [12].

## γδ TCR structure

Human γδ T cells have distinct innate and adaptive functions that are determined by their TCR sequences, which result from somatic recombination. Specifically, the TCR γ and δ loci undergo recombination processes, where Vδ1–3 and Vγ2–5, Vγ8, and Vγ9 genes are key to γδ T cell diversity, resulting in certain chain pairings that are more common in specific tissues. These chain pairings are divided into three major families: Vδ1, Vδ2, and Vδ3. Vδ1 TCRs exhibit extensive diversity, contributing to unique adult repertoires, while Vδ2 T cells, with a semi-invariant repertoire, largely respond to phosphoantigens via interactions with BTN3A1. Vδ3 T cells, with a limited TCR repertoire, are also implicated in potential therapeutic applications due to their unique antigen binding capabilities. Notwithstanding the differences between γδ TCR families, every γδ TCR structure interacts with a CD3 subunit to form an octameric complex, initiating signaling upon antigen engagement.

The distinct functions of human γδ T cells, whether innate or adaptive, are linked to specific cell subsets with different developmental origins, as evidenced by their diverse TCR sequences resulting from somatic recombination [1]. For instance, the TCR δ repertoire in human skin is distinct and restricted from that in the peripheral blood [142]. Comparable to the TCRβ (*TRB*) locus, the TCR δ (*TRD*) locus undergoes recombination of joining (J), diversity (D), and variable (V) segments [1]. There are eight *TRDV* genes (Vδ1–8), with Vδ1–3 being specific to γδ T cells and Vδ4–8 being shared with the TCRα locus [1]. Among the eight Vδ variants, Vδ1, Vδ2, and Vδ3 are notably the most utilized gene segments, thus serving as key markers for classifying γδ T cell families [1, 142–144]. These genes can rearrange with three *TRDD* (D1–3) and four *TRDJ* genes (J1–4) [1]. On the other hand, although the TCRγ (*TRG*) locus, akin to the TCRα (*TRA*) locus, undergoes VJ recombination, only six of the 14 *TRGV* genes are functional (Vγ2–5, Vγ8, and Vγ9), and can recombine with five *TRGJ* genes (J1, J2, JP, JP1, and JP2) [1]. Following recombination, the transcribed and spliced V(D)J gene segments are combined with constant exons (C in *TRD* and either C1 or C2 in *TRG*), resulting in unique γδ TCR heterodimers expressed on the cell surface [1, 145]. While a vast number of distinct γδ TCR sequences (10^17^–10^18^) could theoretically be generated due to V(D)J recombination diversity, certain rearrangements and chain pairings are significantly more prevalent, influenced not only by biases in recombination but also by the selection of functional T cell clonotypes, such as various oligoclonal populations prevailing in different tissues and circulation [1, 62]. Interestingly, deep sequencing of TCR β and TCR γ repertoires suggests that TCR β rearranges after αβ and γδ T cell commitment [146].

**Fig. 4:**
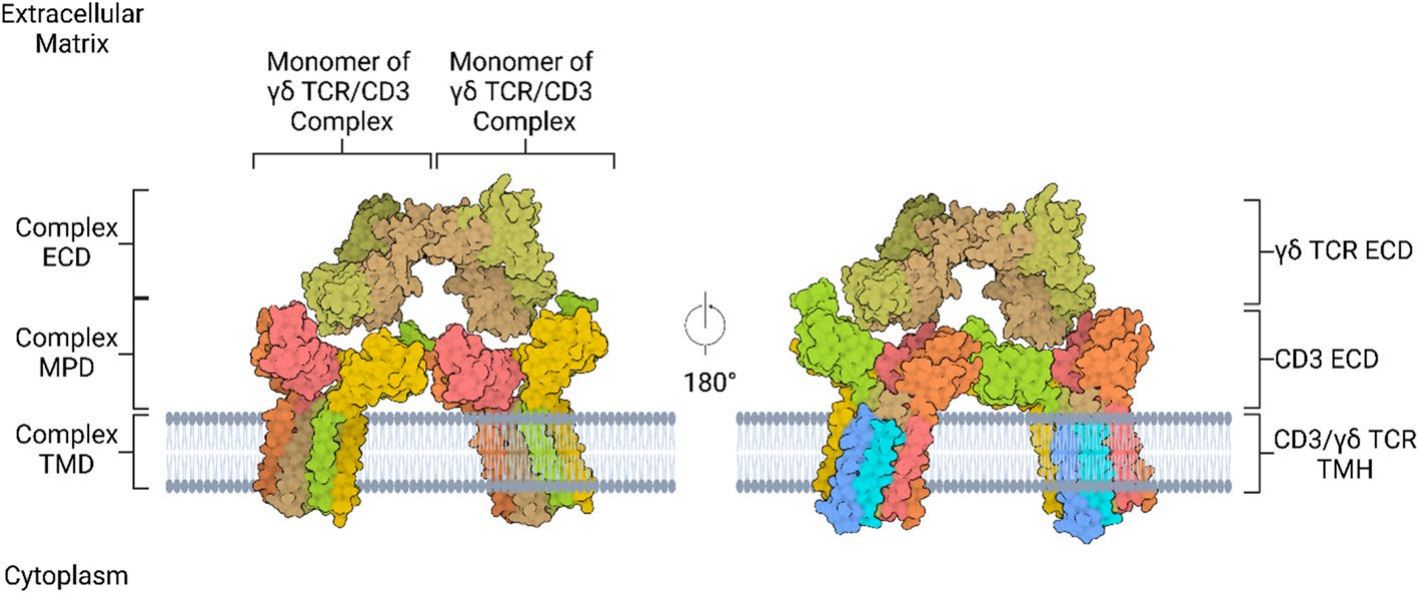
Dimer of γδ TCR/CD3 complexes. γδ TCRs exist in octameric complexes with CD3 proteins; in Vγ2Vδ1 and Vγ5Vδ1 T cells, these octameric complexes form dimers with each other. Most distally from the plasma membrane, the membrane-distal Vγ/Vδ (narrower brown blob/narrower olive-green blob) and membrane-proximal Cγ/Cδ (wider brown blob/wider olive-green blob) subdomains of the γδ TCR ECDs form the ECD of the octameric complex. Closer to the plasma membrane, the MPCPs of the γδ TCR and the CPs of the CD3εγ and CD3ε’δ subunits connect the γδ TCR ECDs to the ECDs of the CD3εγ (CD3ε = light green blob; CD3γ = gold blob) and CD3ε’δ (CD3ε’ = pink blob; CD3δ = orange blob) subunits, which CD3 ECDs form the MPD of the octameric complex. Within the plasma membrane, the TMHs of the γδ TCR (TCRγ = brown helix; TCRδ = olive-green helix) and CD3 subunits CD3εγ (CD3ε = light green helix; CD3γ = gold helix), CD3ε’δ (CD3ε’ = pink helix; CD3δ = orange helix), and CD3ζζ’ (CD3ζ = periwinkle helix; CD3ζ’ = cyan helix) form the TMD of the octameric complex. Intracellularly, the short cytoplasmic tails (various colored helices) of the CD3 subunits contain ITAMs, which are phosphorylated upon antigen recognition by the γδ TCR. CD, cluster of differentiation; TCR, T cell receptor; ECD, extracellular domain; MPCP, membrane-proximal connecting peptide; CP, connecting peptide; MPD, membrane-proximal domain; TMD, transmembrane domain; TMH, transmembrane helix; ITAM, immunoreceptor tyrosine-based activation motif. Created with BioRender.com

A thorough analysis of the human γδ TCR reveals that the γδ TCR structure is every bit as intriguing as the genetic events preceding its expression. Specifically, TCR γ and TCR δ chains interact with three dimeric CD3 subunits – CD3 εγ, CD3 ε’δ, and CD3 ζζ’ [147–149]. These three CD3 subunits and the γδ TCR form an octameric complex [147–149]. Moreover, TCR γ and TCR δ subunits each consist of an extracellular domain (ECD) with membrane-distal Vγ/Vδ and membrane-proximal Cγ/Cδ subdomains, a membrane-proximal connecting peptide (MPCP), a transmembrane helix (TMH), and a short cytoplasmic tail (Fig. 4) [149]. Likewise, not only do CD3 γ, CD3 δ, CD3 ε, and CD3 ε’ subunits each possess a connecting peptide, ECD, and TMH, but also a cytoplasmic immunoreceptor tyrosine-based activation motif (ITAM) (Fig. 4) [149]. Contrarily, CD3 ζ and CD3 ζ’ each lack an ECD and MPCP but have a single TMH and three cytoplasmic ITAMs (Fig. 4) [147, 149]. Together, the ECDs of the γδ TCR form the ECD of the octameric complex; the MPCPs of the γδ TCR and the connecting peptides of the CD3 εγ and CD3 ε’δ subunits connect the γδ TCR ECDs to the ECDs of the CD3 εγ and CD3 ε’δ subunits, which CD3 ECDs form the membrane-proximal domain of the octameric complex; and the TMHs of the γδ TCR and CD3 subunits CD3 εγ, CD3 ε’δ, and CD3 ζζ’ form the transmembrane domain of the octameric complex (Fig. 4) [149]. Upon antigen engagement, the octameric complex initiates phosphorylation of the cytoplasmic ITAMs of the CD3 subunits, facilitating downstream events [149–152]. Although all human γδ TCRs follow general structural and functional patterns, notable structural differences do occur.

In Vδ1 TCRs, the diversity in V(D)J gene rearrangement and pairing of Vγ and Vδ1 chains are more extensive compared to Vδ2 TCRs, with four prevailing Vγ/Vδ combinations: Vγ2Vδ1, Vγ3Vδ1, Vγ4Vδ1, and Vγ5Vδ1 [1, 7, 144, 149]. Whereas Vγ3Vδ1, Vγ4Vδ1, and the uncommon Vγ9Vδ1 TCRs exist as monomers, Vγ2Vδ1 and Vγ5Vδ1 TCRs exist as dimers [149]. In newborns, the Vδ1 TCR repertoire is broad and without prevalence of specific clonotypes, while adults typically exhibit TCR privacy – the phenomenon where specific dominant Vδ1 clones provide a repertoire of TCRs unique to each individual [1, 153, 154]. This privacy is mainly attributed to the *TRD* genes/δ chains, whereas the *TRG* gene/γ chain repertoire tends to be shared among individuals [1, 154, 155]. Consequently, the selection and expansion of long-lived Vδ1 T cells with advantageous TCRs during adulthood resemble a memory-like process, suggesting an adaptive immunosurveillance role [1, 153, 154]. Furthermore, it is generally accepted that TCR ligand recognition facilitates clonotype selection [1]. Notwithstanding private TCR repertoires, degenerate recognition could cause diverse TCRs to react to the same conserved ligands [1]. Vδ1 TCRs can recognize various ligands, including lipid antigens, MICA, MICB, and MHC-like proteins such as CD1c and CD1d, although the in vivo impact of these interactions remains unclear [1, 18, 156–160].

Contrary to Vδ1 T cells, Vδ2 T cells exhibit a semi-invariant TCR repertoire that can be shared among individuals, resulting almost exclusively from the pairing of the Vδ2 and Vγ9 TCR chains; hence, Vδ2 T cells are often referred to as Vγ9Vδ2 cells, although previously, as Vγ2Vδ2 cells [1, 146, 161, 162]. Moreover, Vγ9Vδ2 TCRs are known to exist as monomers and can respond robustly to unconventional antigens called phosphoantigens (Fig. 1), such as non-peptidic prenyl pyrophosphates, which accumulate in malignant or infected cells [1, 149, 163]. Notably, phosphoantigens do not directly interact with Vδ2 TCRs but instead bind to the butyrophilin subfamily 3 member A1 (BTN3A1/CD277) intracellular domain, causing BTN3A1 to undergo a conformational change [1, 43, 164–167]. This change enables BTN3A1 to interact with BTN2A1, which can then bind to the Vδ2 TCR Vγ9 chain (Fig. 3) [1, 168, 169]. However, the recognition of prenyl pyrophosphates is dependent upon all complementarity-determining regions of the TCR [163]. While Vδ2 T cells are well known for responding to non-peptide antigens, their TCRs can also recognize peptide antigens such as MSH2, the α and β subunits of the F1-ATPase, and delipidated APOA1, which serves as a stabilizing bridge between the β subunit of the F1-ATPase and the Vγ9Vδ2 TCR [160, 170, 171]. Interestingly, although the F1 ATPase is a mitochondrial protein and MSH2, a nuclear protein, both are expressed ectopically in various cancer cells [170, 171].

Similar to Vδ2 T cells, Vδ3 T cells also exist as monomers and exhibit a semi-invariant TCR repertoire that can be shared among individuals, albeit resulting almost exclusively from the pairing of the Vδ3 and Vγ8 TCR chains [1, 149]. Consequently, Vδ3 T cells are often called Vγ8Vδ3 cells [1]. Additionally, as previously mentioned, Vδ3 TCRs can bind to areas outside the antigen binding cleft of some antigens, indicating possible therapeutic uses [1, 60, 61]. Thus, understanding the specific TCR clonotypes that recognize malignant antigens is crucial for optimizing synthetic TCR engineering in immunotherapy [1].

## Standard γδ T cell therapies

For almost 20 years, γδ T cells have been used in experimental cancer therapies, either by activating in vivo γδ T cells or through the administration of Vδ2-enriched autologous or allogenic peripheral blood mononuclear cells (PBMCs), often combined with other adjunct treatments. The most widely studied therapy involves activating in vivo γδ T cells with zoledronate and/or IL-2, showing positive results in various cancers, including prostate, breast, and melanoma, with Vδ2 T cells expanding at tumor sites. Another approach combines zoledronate and IL-2 with engraftment of Vδ2-enriched PBMCs, used for solid cancers like colorectal and pancreatic cancers – although, results have been mixed – with some patients showing tumor progression despite localized reductions. Additionally, phosphoantigens, such as 2-methyl-3-butenyl-1-pyrophosphate (2M3B1PP), have been used to expand γδ T cells, yielding promising results in extending tumor doubling times; nevertheless, some compounds like bromohydrin pyrophosphate (BrHPP) caused adverse effects. Other methods, such as using irreversible electroporation or anti-BTN3A antibodies, have also been tested, with some success in improving survival and enhancing Vδ2 T cell activity at tumor sites.

Since 2007, γδ T cells have been used in various experimental cancer therapies, either through the targeting of in vivo γδ T cells with stimulators and/or the administration of γδ T cells as co-treatments with adjunct therapies (summarized in Table 1) [12]. Of these therapies, the most widely studied is the use of in vivo γδ T cells activated with zoledronate and/or IL-2, with or without letrozole as an adjunct, for use in prostate, renal cell, and breast cancers, as well as melanoma and neuroblastoma [12, 172–178]. This regimen was well tolerated, resulting in better expansion and maintenance of Vδ2 T cells with less Vδ2 T cells found in circulation [12, 172–178]. Potentially, reduced levels of Vδ2 T cells in circulation denotes higher levels of Vδ2 T cells at tumor sites [12]. The second most commonly studied therapy involves combining the stimulation of in vivo γδ T cells using zoledronate and IL-2 with the subsequent engraftment of Vδ2-enriched autologous PBMCs, with or without zoledronate or gemcitabine as an adjunct, to combat mixed solid cancers like colorectal, gastric, pancreatic, and non-small cell lung cancers [12, 179–186]. Although this treatment model resulted in elevated plasma IFN-γ and increased Vδ2 T cell activity at tumor sites, only one patient showed partial reductions of lung lesions and two patients showed reductions of local ascites, with all three of these patients experiencing new metastases at distant sites [12, 179–186]. Whereas some zoledronate-supplemented therapies were tested on multiple different cancers, others were specific to one or two cancer types [12]. For example, in vivo γδ T cells expanded with zoledronate and IL-2 followed by infusion of Vδ2-enriched autologous PBMCs, using autologous expanded NK and αβ T cells post–radiofrequency ablation as an adjunct, yielded an improvement in progression-free survival in hepatocellular carcinoma patients [12, 187]. Another example involves the use of in vivo γδ T cells activated with zoledronate and various cytokines, as well as subsequent Vδ2-enriched allogenic PBMC engraftment, which was well tolerated in cholangiocarcinoma patients [12, 188]. However, stimulation of in vivo γδ T cells with zoledronate, IL-2, IL-15, and vitamin C followed by infusion of Vδ2-enriched allogenic PBMCs, using irreversible electroporation, iodine-125, and/or cryoablation as adjunct(s) to treat lung and liver cancers resulted in only one case of complete response, with this complete response coming from a patient who received the iodine-125 adjunct therapy [12, 189].

**Table 1:** Standard γδ T cell therapies.

Year(s)	Technology	Cancer(s)	Effects/Status	Citation(s)
2007, 2011	2M3B1PP and IL-2 followed by Vδ2-enriched autologous PBMC adoptive cell therapy with/without zoledronate​	Renal cell carcinoma	Tumor doubling time prolonged	[12, 192, 193]
2007, 2010–2013, 2016, 2018	Zoledronate with/without IL-2, with/without letrozole	Prostate, breast, and renal cell cancers; melanoma; and neuroblastoma​	Well tolerated, less Vδ2 γδ T cells in blood (potentially more at tumor), better expansion and maintenance of Vδ2 γδ T cells ​	[12, 172–178]
2008, 2010	BrHPP and IL-2 followed by nothing or Vδ2-enriched autologous PBMC adoptive cell therapy	Renal cell carcinoma and mixed solid cancers​	Tachyphylaxis with repeated dosing, disseminated intravascular coagulation	[12, 190, 191]
2010–2011, 2013–2014, 2017, 2020	Zoledronate and IL-2 followed by Vδ2-enriched autologous PBMC adoptive cell therapy with/without gemcitabine​	Non-small cell lung, colorectal, gastric, pancreatic, and mixed solid cancers	Elevated plasma IFN-γ; increased Vδ2 T cell activity at tumor site; partial reductions of lung lesions (1 patient) and local ascites (2 patients), all with new metastases​	[12, 179–186]
2013	Zoledronate and IL-2 followed by Vδ2-enriched autologous PBMC adoptive cell therapy with autologous expanded NK cells and αβ T cells at unspecified ratio post–radiofrequency ablation​	Hepatocellular carcinoma	Improvement in​ progression-free survival	[12, 187]
2019	Zoledronate and undisclosed cytokines followed by Vδ2-enriched allogenic PBMC adoptive cell therapy	Cholangiocarcinoma	Well tolerated	[12, 188]
2020	Zoledronate and IL-2 followed by Vδ2-enriched allogenic PBMC adoptive cell therapy with irreversible electroporation​	Pancreatic cancer	Modest improvement in survival when Vδ2 treatment is followed by irreversible electroporation​	[12, 194]
2021	Zoledronate, IL-2, IL-15, and vitamin C followed by Vδ2-enriched allogenic PBMC adoptive cell therapy with irreversible electroporation, iodine-125, and/or cryoablation​	Lung and liver​ cancers	One case of complete response in patient who also had concurrent iodine-125 therapy​	[12, 189]
2021	Anti-BTN3A agonist antibody​	Mixed solid cancers​	Less Vδ2 T cells in blood (potentially more at tumor)​	[12, 42]
2023	Gastric cancer exosomal THBS1 to enhance production of IFN-γ, TNF-α, perforin, and granzyme B in vitro, and elevate killing of gastric cancer cells by Vδ2 T cells in vivo	Gastric cancer	Successful preclinical trials​	[195, 196]

*2M3B1PP *2-methyl-3-butenyl-1-pyrophosphate, *PBMC *peripheral blood mononuclear cell, *BrHPP *bromohydrin pyrophosphate, *IL *interleukin, *NK *natural killer, *BTN3A *butyrophilin subfamily 3 member A1, *THBS1 *thrombospondin 1, *IFN *interferon, *TNF *tumor necrosis factor

Additionally, phosphoantigens have been used to expand in vivo γδ T cells for uses in treating renal cell carcinoma and other solid tumor cancers [12, 89, 190–193]. Specifically, administration of 2M3B1PP and IL-2 preceding Vδ2-enriched autologous PBMC adoptive cell therapy, with low-dose IL-2 and/or zoledronate as adjunct(s), facilitated prolongation of tumor doubling time [12, 192, 193]. Interestingly, BrHPP and IL-2, followed by either no additional treatment or Vδ2-enriched autologous PBMC adoptive cell therapy with low-dose IL-2 as an adjunct only caused adverse effects, such as disseminated intravascular coagulation or tachyphylaxis with repeated dosing [12, 190, 191].

In other cases, neither zoledronate, IL-2, nor phosphoantigens were used to activate in vivo γδ T cells. For instance, irreversible electroporation followed by adoptive cell therapy with Vδ2-enriched allogenic PBMCs facilitated a modest improvement in survival for pancreatic cancer patients [12, 194]. Also, the use of anti-BTN3A agonist antibodies to combat various solid tumors resulted in less circulating Vδ2 T cells (again, suggesting the attraction of more Vδ2 T cells to the tumor sites) [12, 42]. Furthermore, the use of gastric cancer exosomal THBS1 to enhance production of IFN-γ, TNF-α, perforin, and granzyme B in vitro, to ultimately elevate the killing of gastric cancer cells by Vδ2 T cells in vivo, has yielded successful preclinical trials [195, 196].

## Non-CAR γδ T cell engineering

Engineered γδ T cells have been developed to target specific cancers, including both non–chimeric antigen receptor (non-CAR)- and chimeric antigen receptor (CAR)-based approaches. One early non-CAR method used polysaccharide K (PSK) to stimulate γδ T cells, showing anti-tumor effects in HER2^+^ breast cancer models. In subsequent years, other non-CAR approaches enhanced γδ T cell efficacy, such as inhibiting CD3ε for better tumor killing and using monoclonal antibodies against CTLA-4 and PD-1 to treat melanoma, yielding positive preclinical results. More recently, non-CAR γδ T cells targeting peripheral blood cancers were developed, such as an anti-CD19 non-CAR γδ T cell, which showed strong responses in lymphoma treatment without significant immune exhaustion. The latest innovation, which involves engineering Vδ2 T cells to produce synthetic opsonins, targets osteosarcoma with enhanced cytotoxicity, improves tumor control, and reduces persistence in blood, thus showing promise for osteosarcoma therapy.

Since over a decade, there has been a drive to engineer enhanced γδ T cells specific to certain types of cancers. Among these engineered γδ T cells are non-CAR γδ T cells, which are typically made via expansion followed by stimulation or inhibition of certain γδ T cell surface markers, as opposed to the engineering of a CAR (summarized in Table 2). One of the first types of these non-CAR γδ T cells was developed in 2013 at the University of Washington [197]. In this study, expanded γδ T cells were stimulated with PSK from the *Trametes versicolor* (turkey tail) mushroom, which resulted in IFN-γ production and upregulation of CD25, CD69, and CD107a [197, 198]. Moreover, these PSK-stimulated γδ T cells reciprocally activated dendritic cells and in the absence of dendritic cells, co-activated other γδ T cells with TCR cross-linking in vitro [197]. Furthermore, PSK treatment stimulated γδ T cells among tumor-infiltrating lymphocytes in vivo, contributing to the anti-tumor effects of PSK [197]. Overall, these non-CAR γδ T cells were successful in both in vitro and in vivo preclinical trials against HER2^+^ breast cancer [197]. Another non-CAR γδ T cell type was engineered in 2014 and 2018 by inhibiting CD3ε on expanded Vδ2 T cells with anti-CD3ε antibodies [199, 200]. The inhibition of CD3ε enhanced γδ TCR signaling through stabilization of the active CD3 conformation, as well as activation and tumor-killing efficacy of these γδ T cells – by an unknown mechanism, although independent of Nck recruitment to the γδ TCR – leading to an increased γδ T cell–mediated release of cytotoxic granules and cancer cell lysis, thus denoting successful in vitro and in vivo preclinical trials against B-cell lymphoma and pancreatic ductal adenocarcinoma [199, 200]. Additionally, in 2022, a non-CAR γδ T cell was developed via the inhibition of CTLA-4 and PD-1 on Vδ2 T cells using monoclonal antibodies to increase γδ T cell infiltration and killing of melanoma cells [201]. This study yielded successful preclinical trials [201]. Compared to Vδ2 T cells engineered with the isotype control (IgG) antibody, anti CTLA-4/anti PD-1 Vδ2 T cells showed a marked increase in melanoma spheroid infiltration and reduction of spheroid volume, indicating an improved killing efficacy of melanoma cells [201].

**Table 2:** Engineered non-CAR γδ T cell therapies

Year(s)	Technology	Cancer(s)	Effects/Status	Citation(s)
2013	Stimulation of IFN-γ and upregulation of CD25, CD69, and CD107a in γδ T cells using polysaccharide K from mushrooms​	HER2^+^ breast cancer	Successful in vitro and in vivo preclinical trials	[197]
2014, 2018	Inhibition of CD3ε on Vδ2 T cells with anti-CD3ε antibodies	B-cell lymphoma and pancreatic ductal adenocarcinoma	Successful in vitro and in vivo preclinical trials	[199, 200]
2018, 2023	Engineered human anti-CD19 antibody and fused its Fab fragment to γδ TCR constant chain with adding scFv/CD28 co-stimulatory molecule to anti-CD19 fragment	Relapsed/refractory diffuse large B-cell and primary CNS lymphomas	Successful preclinical and phase 1 clinical trials; induced rapid complete responses and durable remissions; good safety profile	[202, 203]
2022	Inhibition of CTLA-4 and PD-1 on Vδ2 T cells using mAbs to increase γδ T cell infiltration and killing of melanoma cells​	Melanoma	Successful in preclinical trials	[201]
2023	CD20-directed Vδ2 T cells (generated using antibody-cell conjugation)	Relapsed/refractory B-cell lymphoma	Successful in vitro and in vivo preclinical trials	[204]
2024	Vδ2 T cells modified to secrete stIL15 and scFv-Fc fusion proteins targeting GD2 (stIL15-OPS-γδ T cells)	Osteosarcoma	Successful in vitro and in vivo preclinical trials	[205]

*IFN *interferon, *CD *cluster of differentiation, *scFv *single-chain variable fragment, *Fc *crystallizable fragment, *CTLA-4 *cytotoxic T lymphocyte–associated protein 4, *PD-1 *programmed cell death protein 1, *mAb *monoclonal antibody, *stIL15 *interleukin-15 receptor α–interleukin-15, *OPS *opsonin, *GD2 *ganglioside G2

More recently, the development of non-CAR γδ T cells has been more focused on peripheral blood cancers. For example, in 2023, a special non-CAR γδ T cell was engineered where the F_ab_ fragment of a human anti-CD19 antibody was fused to the γδ TCR constant chain with the addition of the single-chain variable fragment (scFv) of the anti-CD19 antibody and a CD28 co-stimulatory molecule to the anti-CD19 F_ab_ fragment [202, 203]. This anti-CD19 non-CAR γδ T cell was successful in combating relapsed/refractory diffuse large B-cell lymphoma and primary CNS lymphoma [202, 203]. Specifically, preclinical and phase 1 clinical trials reported that this anti-CD19 non-CAR γδ T cell induced rapid complete responses and durable remissions with a good safety profile [202, 203]. Interestingly, the anti-CD19 non-CAR yielded the same efficacy but reduced exhaustion markers, such as PD-1, LAG-3, and TIM-3, and cytokine release compared with the anti-CD19 CAR [202, 203]. The decreases in cytokine release and immune exhaustion markers while maintaining tumor-killing efficacy is ideal, as such indicates a milder immune response. Additionally, in 2023, CD20-directed Vδ2 T cells were generated using antibody-cell conjugation (ACC) with rituximab, as opposed to using CAR technology [204]. These anti-CD20 γδ T cells were successful in both in vitro and in vivo preclinical trials against relapsed/refractory B-cell lymphoma [204]. In this case, T cell activation and increased cytotoxicity against CD20-expressing cancer cells was achieved through antigen recognition of the ACC-linked anti-CD20 antibody [204].

An exciting and most recent development involves the engineering of Vδ2 T cells to produce synthetic opsonins in the form of mitogenic IL-15Rα–IL-15 (stIL15) and scFv–Fc fusion proteins [205]. Specifically, these γδ T cells were designed to secrete opsonins targeting GD2 (stIL15–OPS–γδ T cells) for treating osteosarcoma [205]. stIL15–OPS–γδ T cells promote bystander activity of myeloid and other lymphoid cells, while exhibiting enhanced cytotoxicity independent of transgene expression [205]. Compared to unmodified γδ T cells, stIL15–OPS–γδ T cells facilitate improved in vivo control of subcutaneous osteosarcoma tumors as well as reduced osteosarcoma persistence in the blood [205]. Moreover, whereas stIL15–OPS–γδ T cells were effective against patient-derived osteosarcomas both in vitro and in vivo, efficacy was enhanced with the addition of zoledronate [205]. Notably, stIL15–OPS–γδ T cells favor osteosarcoma-homing and ADCC, recruiting other immune cells to promote antibody-dependent cellular phagocytosis and ADCC against antigen-positive tumor cells [205]. Taken together, by combining bystander activation and direct cytolysis, stIL15–OPS–γδ T cells serve as a promising allogeneic cell therapy to combat osteosarcoma [205].

## CAR γδ T cell engineering

CAR γδ T cells have been engineered to target specific cancers, incorporating CARs for both activation and antigen binding. For example, glypican-3–specific CAR Vδ1 T cells with co-expressed IL-15 demonstrated enhanced anti-tumor activity against hepatocellular carcinoma, showing robust expansion and in vivo efficacy. Moreover, engineered anti-CD20 CAR Vδ1 T cells yielded high response rates and a favorable safety profile in clinical trials against lymphoma, with no significant GvHD. Another advancement was the creation of anti-mesothelin CAR Vδ2 T cells, which showed effective targeting of ovarian cancer both in vitro and in vivo, with increased IL-15 expression further enhancing anti-tumor effects. More recently, the development of anti-αvβ6 CAR G115 Vδ2 T cells for pancreatic, triple-negative breast cancers, and chronic myelogenous leukemia demonstrated promising dual-specific cancer immunotherapy potential, while anti-CD22 CAR γδ T cells proved effective in eradicating B-cell acute lymphoblastic leukemia (B-ALL).

In the past decade, there also has been a drive to develop CAR γδ T cells. These engineered γδ T cells have chimeric antigen receptors, which denotes receptors with both activation and antigen-binding functions. As is the case with some non-CAR γδ T cells, there are CAR γδ T cells that have been engineered to be specific for certain types of cancers (summarized in Table 3). For example, in 2021, Vδ1 T cells were engineered using a glypican-3–specific CAR and soluble IL-15 to combat hepatocellular carcinoma [206, 207]. These anti–glypican-3 CAR Vδ1 T cells co-expressing secreted IL-15 displayed robust expansion from PBMCs [206, 207]. Upon expansion, these anti–glypican-3 CAR Vδ1 cells exhibited minimal inhibitory receptor expression manifested by robust in vitro anti-tumor activity against hepatocellular carcinoma cells, even when in the presence of soluble glypican-3 [206, 207]. Furthermore, co-expression of secreted IL-15 increased proliferation and maintained long-term cytotoxicity of these anti–glypican-3 CAR Vδ1 T cells [206, 207]. Importantly, anti–glypican-3 CAR Vδ1 cells with secreted IL-15 co-expression augmented in vivo anti-tumor activity compared to those without secreted IL-15 co-expression [206, 207]. Neither case resulted in xenogeneic GvHD [206, 207].

**Table 3:** Engineered CAR γδ T cell therapies

Year(s)	Technology	Cancer(s)	Effects/Status	Citation(s)
2021	Vδ1 T cells engineered using glypican-3–specific CAR and soluble IL-15​	Hepatocellular carcinoma	Successful preclinical in vitro and in vivo studies​	[206, 207]
2022	CD20-directed CAR–Vδ1 T cells​	Diffuse large B-cell, large B-cell, high-grade B-cell, mantle cell, and Burkitt lymphomas	Successful preclinical and phase 1 clinical trials	[208, 209]
2023	Increasing CD16 on Vδ2 T cells to enhance ADCC using anti-mesothelin CAR engineering with or without increased IL-15 expression	Ovarian cancer	Successful preclinical in vitro and in vivo studies (even better with increased IL-15 expression)	[210]
2024	Modification of G115 γδ TCR to recognize αvβ6-expressing tumor cells (CAR Vδ2 T cell)​	Triple-negative breast and pancreatic cancers	Successful in vitro preclinical trial	[211, 212]
2024	γδ anti-CD22 CAR T cell (both Vδ1 and Vδ2)​	B-cell acute lymphoblastic leukemia	Completely successful preclinical trials; now in clinical trials	[213]

*CAR, *chimeric antigen receptor, *IL *interleukin, *CD *cluster of differentiation, *ADCC *antibody-dependent cellular cytotoxicity, *TCR *T cell receptor

A second example of engineered, anti-cancer CAR γδ T cells is the CD20-directed CAR Vδ1 T cell, designed in 2022 for combating various lymphomas, such as large B-cell, diffuse large B-cell, high-grade B-cell, mantle cell, and Burkitt lymphomas [208, 209]. This anti-CD20 CAR Vδ1 T cell was engineered using a novel, fully human anti-CD20 monoclonal antibody and expresses multiple chemokine receptors and NKRs [209]. Moreover, these experimental anti-CD20 CAR Vδ1 cells exhibited anti-tumor activity both in vitro and in vivo during preclinical trials, without any xenogeneic GvHD [209]. Furthermore, in a phase 1 clinical trial of 11 patients, one patient developed grade 1 cytokine release syndrome and another, grade 2 [208]. A third patient developed grade 1 immune effector cell-associated neurotoxicity syndrome; however, this adverse effect resolved within 24 hours [208]. Additionally, there were no dose-limiting toxicity or GvHD events [208]. The best objective response rate (ORR) and CR rate were 78% [208]. Of the four patients who received prior anti-CD19 CAR T cell therapies, both the ORR and CR rate were 100% [208]. Upon completion of the trial, of the seven patients who achieved CR, one died while in complete remission, two progressed, and four were still in CR [208]. Overall, the anti-CD20 CAR Vδ1 T cells showed a favorable safety profile, an encouraging CR rate, and sustained durability [208].

Another example of an anti-cancer CAR γδ T cell is the anti-mesothelin CAR Vδ2 T cell enhanced with increased CD16 expression to augment ADCC, with or without additional increased IL-15 expression, for targeting ovarian cancers [210]. These CAR γδ T cells exhibit pronounced in vitro anti-tumor activity against multiple ovarian cancer lines and can kill tumor-associated macrophages [210]. Moreover, anti-mesothelin CAR Vδ2 T cells are efficacious and safe in subcutaneous and intraperitoneal in vivo ovarian cancer models [210]. Interestingly, these anti-mesothelin CAR γδ T cells perform even better when IL-15 expression is increased, leading to profound anti-tumor effects both in vitro and in vivo [210].

Most recently, an anti-αvβ6 CAR G115 Vδ2 T cell was developed with a modification in the γδ TCR to recognize αvβ6- and phosphoantigen-expressing tumor cells, such as those found in pancreatic and triple-negative breast cancers, as well as chronic myelogenous leukemia (CML), respectively [211, 212]. G115 is a clonal Vδ2 TCR that can confer responsiveness to phosphoantigens when genetically inserted into αβ T cell genomes [211]. To broaden the cancer specificity of G115, a tumor-binding dodecamer peptide was selected from the foot-and-mouth disease virus and inserted into the complementarity-determining region 3 of the TCR δ2 chain [211]. This dodecamer peptide, known as the A20 peptide, binds with high selectivity and affinity to the epithelial-selective integrin αvβ6, which is expressed in various solid tumors [211]. Consequently, the anti-αvβ6 CAR G115 Vδ2 T cell is able to kill both αvβ6- and phosphoantigen-expressing tumor cells, with enhanced cytolytic activity against K562 CML cells [211, 212]. Moreover, anti-αvβ6 CAR G115 Vδ2 cell activation resulted in IFN-γ release in the presence of either αvβ6 or phosphoantigen [211]. Thus, given these successful in vitro preclinical trials, anti-αvβ6 CAR G115 Vδ2 T cells show promise as a novel dual-specific cancer immunotherapy [211].

An additional most recent development involves the anti-CD22 CAR γδ T cell for the treatment of B-ALL [213]. Interestingly, there was a major difference in performance based on whether these anti-CD22 CAR γδ T cells were purified via positive or negative selection [213]. Specifically, positive selection resulted in a significantly increased in vitro secretion of IL-2 and IFN-γ but a decreased in vitro tumor killing rate [213]. Conversely, negative selection resulted in reduced cytokine levels in response to CD22^+^ B-ALL, but a quicker ex vivo tumor killing rate with significant enrichment of anti-CD22 CAR Vδ1 and Vδ2 T cells [213]. Nevertheless, whether purified through positive or negative selection, anti-CD22 CAR γδ T cells exhibited similar in vivo killing efficacy against B-ALL cells [213]. Furthermore, when anti-CD22 CAR γδ T cells were developed with both positive and negative selection, the B-ALL cells were efficiently eradicated upon treatment [213].

## Corporate clinical trials

At least eight pharmaceutical companies are currently conducting clinical trials to test engineered γδ T cell therapies for cancer treatment. Takeda Pharmaceutical Company is exploring allogeneic Vδ1 T cell therapies for acute myeloid leukemia, which are currently in phase 1 clinical trials. IN8bio is testing various γδ T cell technologies, including genetically modified autologous and allogeneic therapies for glioblastoma and leukemia, with some in phase 1 and phase 2 clinical trials. Acepodia is developing anti-CD20 and anti-EGFR γδ T cell therapies for various cancers, which are presently in phase 1 clinical trials. Other companies, such as TC BioPharm, CytoMed Therapeutics, Kiromic Biopharma, Lava Therapeutics, and Adicet Bio, are advancing various γδ T cell therapies, including but not limited to CAR-engineered therapies targeting solid and hematological cancers, with multiple therapies progressing through phase 1, 2, and 3 clinical trials.

Although some clinical trials have been previously discussed in this review, this section describes clinical trials conducted strictly by pharmaceutical companies, the specific details of which are undisclosed due to the necessity of maintaining trade secrets. There are currently at least eight companies that have engineered and are clinically testing γδ T cells for the purpose of treating cancer (summarized in Table 4). One of these companies – the Tokyo- and Osaka-based Takeda Pharmaceutical Company Ltd. – which acquired GammaDelta Therapeutics Ltd. in 2021 and Adaptate Biotherapeutics in 2022, is testing allogeneic Vδ1 T cell therapy platforms, including both blood-derived and tissue-derived platforms, as well as early-stage cell therapy programs for the treatment of relapsed/refractory acute myeloid leukemia [214–216]. Currently, these experimental therapies are in phase 1 clinical trials [216].

**Table 4:** Corporate clinical trials for γδ T cell therapies

Company	Technology Pipeline	Cancer(s)	Citation(s)
Takeda Pharmaceutical Company Ltd.^a^	Allogeneic Vδ1 T cell therapy platforms, including both blood-derived and tissue-derived platforms, as well as early-stage cell therapy programs (phase 1 clinical trials)​	Relapsed/refractory acute myeloid leukemia	[214–216]
IN8bio	DeltEx DRI Auto (INB-200) (phase 1 clinical trials)​ DeltEx Allo (phase 1 clinical trials)​ DeltEx DRI Auto (INB-400) (phase 2 clinical trials)​	Glioblastoma and leukemia	[217]
Acepodia	CD20-targeting, ACC-γδ2 T cell therapy (phase 1 clinical trials)​ EGFR-targeting, ACC-γδ2 T cell therapy (entering clinical trials)​	Chronic lymphocytic leukemia; diffuse large B-cell, follicular, and mantle cell lymphomas; and colorectal, non-small cell lung, and triple-negative breast cancers	[218]
TC BioPharm^b^	Unmodified, allogeneic γδ T cells (finished phase 1 clinical trials)​	Acute myeloid leukemia	[219]
CytoMed Therapeutics Ltd	NKG2D–CAR γδ T cell therapy (phase 1 clinical trials)​ Unmodified γδ T cell therapy (clinical trial application)​	Relapsed/refractory solid tumor cancers	[220]
Kiromic Biopharma	Universal, non-engineered γδ T cell therapy in combination with standard anti-tumor modality) (phase 1 clinical trials began Q4 2022)​ Anti–PD-L1 CAR γδ T cell therapy in combination with standard anti-tumor modality or as stand-alone (phase 2 clinical trials began Q2 2023) ​ Anti-mesothelin CAR γδ T cell therapy in combination with standard anti-tumor modality or as stand-alone (began phase 3 clinical trials Q4 2023)​	Solid tumor cancers	[221]
Adicet Bio	Anti-CD20 CAR γδ T cell (phase 1 clinical trials ongoing)​	Relapsed/refractory non-Hodgkin and mantle cell lymphomas	[222]
Lava Therapeutics^c,d^	Anti-PSMA Vδ2 T cell (phase 1 clinical trials)​ Anti-EGFR Vδ2 T cell (phase 1 clinical trials)​	Metastatic castration-resistant prostate and solid tumor cancers	[223]

*DRI *drug-resistant immunotherapy, *CD *cluster of differentiation, *ACC *antibody-cell conjugation, *EGFR *epidermal growth factor receptor, *NKG2D *natural killer group 2D, *CAR *chimeric antigen receptor, *Q *quarter, *PD-L1 *programmed cell death protein ligand 1, *PSMA *prostate-specific membrane antigen

^a^ Acquired GammaDelta Therapeutics Ltd. in 2021 and Adaptate Biotherapeutics in 2022

^b^ Partnered with ÚHKT, Institute of Hematology and Blood Transfusion (Prague, Czech Republic)​

^c^ Clinical collaboration with Merck & Co. as of January 25, 2024​ for anti-PSMA Vδ2 T cell

^d^ Partnered with Pfizer for anti-EGFR Vδ2 T cell

Another company, IN8bio, which is based in Birmingham, Alabama and New York, New York, has multiple experimental γδ T cell technologies undergoing clinical trials [217]. For example, DeltEx Drug Resistant Immunotherapy (DRI) Auto (INB-200) is an autologous, genetically-modified γδ T cell designed for combating glioblastoma and is in phase 1 clinical trials [217]. DeltEx Allo is an allogenic, donor-derived γδ T cell that is administered to patients following hematopoietic bone marrow transplantation for combating leukemia, which is also in phase 1 clinical trials [217]. Similar to DeltEx DRI Auto (INB-200), DeltEx DRI Auto (INB-400) is likewise an autologous, genetically-modified γδ T cell used for combating glioblastoma, but is specifically modified to be resistant to alkylating chemotherapy and is in phase 2 clinical trials [217].

Moreover, Acepodia, which is based in Alameda, California and Taiwan, has engineered a CD20-targeting, ACC Vδ2 T cell therapy for treating chronic lymphocytic leukemia and diffuse large B-cell, follicular, and mantle cell lymphomas [218]. This anti-CD20, ACC Vδ2 T cell is currently in phase 1 clinical trials [218]. Another experimental therapy undergoing testing by Acepodia is the epidermal growth factor receptor (EGFR)-targeting, ACC Vδ2 T cell therapy for use in combating colorectal, non-small cell lung, and triple-negative breast cancers [218]. At present, this therapy is entering clinical trials [218].

Furthermore, the Scotland-based TC BioPharm has developed unmodified, allogeneic γδ T cells for treating acute myeloid leukemia [219]. This therapy was designed in partnership with the Czechia-based ÚHKT (Institute of Hematology and Blood Transfusion) and has finished phase 1 clinical trials [219]. Additionally, the Singapore- and Malaysia-based CytoMed Therapeutics Ltd. has engineered a NKG2D CAR γδ T cell therapy, which is in phase 1 clinical trials, and an unmodified γδ T cell therapy, which is under application for clinical trials [220]. Both of these therapies are designed to target relapsed/refractory solid tumors [220].

Whereas most companies focus on a mix of solid tumor and hematological cancers, Kiromic Biopharma, based in Houston, Texas, focuses solely on off-the-shelf, allogenic γδ T cell therapies for solid tumor cancers, as these cancers comprise around 90% of all cancers [221]. For instance, Deltacel is a universal, non-engineered, γδ T cell therapy designed to be used in combination with a standard anti-tumor modality [221]. Phase 1 clinical trials for Deltacel began in the fourth quarter of 2022 [221]. Procel, an anti PD-L1 CAR γδ T cell technology, was designed to be used as a stand-alone treatment as well as in combination with a standard anti-tumor modality, and began phase 2 clinical trials in the second quarter of 2023 [221]. Another therapy intended for use either in combination with a standard anti-tumor modality or as a stand-alone treatment is Isocel, which is an anti-mesothelin CAR γδ T cell therapy that began phase 3 clinical trials during the fourth quarter of 2023 [221].

Additionally, Lava Therapeutics, based in Philadelphia, Pennsylvania and the Netherlands, has developed an anti-PSMA (prostate-specific membrane antigen) Vδ2 T cell for combating metastatic castration-resistant prostate cancer [222]. This experimental therapy is in phase 1 clinical trials and is marked by an ongoing clinical collaboration with Merck & Co. as of January 25, 2024 [222]. Another therapy undergoing phase 1 clinical trials is the anti-EGFR Vδ2 T cell for the treatment of solid tumors [222]. These clinical trials are being conducted under a partnership with Pfizer [222]. A different direction is currently being explored by Adicet Bio, which is based in Redwood City, California and Boston, Massachusetts [223]. Adicet Bio is using an anti-CD20 CAR γδ T cell for mantle cell lymphoma, which is currently in phase 1 clinical trials [223]. Moreover, this anti-CD20 CAR γδ T cell has been granted Fast Track designation by the United States Food and Drug Administration for clinical testing as a treatment for relapsed/refractory B-cell non-Hodgkin lymphoma [223].

## Conclusions and future perspectives

In conclusion, the vast body of evidence presented in this review underscores the pivotal role of γδ T cells as indispensable orchestrators of immune responses. From diverse tissue distribution to multifunctional capabilities, γδ T cells stand out as key players in immune surveillance and regulation. By delving into the intricate biology of γδ T cells, we have gained valuable insights into the contributions of γδ T cells to human health and the combating of disease, as well as potential targets for therapeutic interventions.

However, despite the significant progress made in elucidating the roles of γδ T cells, several challenges remain to be overcome, presenting exciting avenues for future research. One of the challenges currently facing researchers is that γδ T cell co-treatment effectiveness is still somewhat unknown and shows mixed results. For example, these treatments are, at best, only partially effective and at worst, either ineffective or laced with dangerous side effects. Another challenge is that the stimulation or inhibition of γδ T cell surface markers to enhance γδ T cell performance is not specific, which, if not carefully executed, may result in autoimmune problems. Additionally, to generate cancer-specific γδ T cells, one must find unique cancer cellular surface antigens that are absent on non-cancer cells. This process is challenging because of its time-consuming nature and high monetary costs, as these antigens must be thoroughly investigated for every type and subtype of cancer; otherwise, severe adverse effects may occur. Furthermore, any treatment method that targets a protein on a cancer cell risks the challenge of the cancer cell gaining resistance to that treatment.

Future research directions should focus on addressing the above-mentioned challenges. Addressing these challenges will answer important questions, such as how to develop cancer-specific γδ T cells safely, efficiently, and economically, to provide affordable solutions with negligible risk to any cancer patient. As we continue to unravel the complexities of γδ T cell biology, we anticipate that our expanding knowledge will fuel the development of innovative immunotherapeutic strategies, ultimately benefiting patients across a spectrum of diseases, especially cancer.

## Data Availability

No datasets were generated or analysed during the current study.
